# Characterization of the Upper Respiratory Bacterial Microbiome in Critically Ill COVID-19 Patients

**DOI:** 10.3390/biomedicines10050982

**Published:** 2022-04-23

**Authors:** Xiangning Bai, Aswathy Narayanan, Magdalena Skagerberg, Rafael Ceña-Diez, Christian G. Giske, Kristoffer Strålin, Anders Sönnerborg

**Affiliations:** 1 Department of Laboratory Medicine, Division of Clinical Microbiology, ANA Futura, Karolinska Institute, 141 52 Stockholm, Sweden; christian.giske@ki.se (C.G.G.); anders.sonnerborg@ki.se (A.S.); 2 State Key Laboratory of Infectious Disease Prevention and Control, National Institute for Communicable Disease Control and Prevention, Chinese Center for Disease Control and Prevention, Beijing 102206, China; 3 Division of Laboratory Medicine, Oslo University Hospital, 0372 Oslo, Norway; 4 Department of Medicine Huddinge, Division of Infectious Diseases, Karolinska Institute, 141 86 Stockholm, Sweden; aswathy.narayanan@ki.se (A.N.); rafael.cena.diez@ki.se (R.C.-D.); kristoffer.stralin@sll.se (K.S.); 5 Department of Infectious Diseases, Karolinska University Hospital, 141 86 Stockholm, Sweden; magdalena.skagerberg@sll.se; 6 Department of Clinical Microbiology, Karolinska University Hospital, 171 76 Stockholm, Sweden

**Keywords:** SARS-CoV-2, COVID-19, microbiome, upper respiratory tract, respiratory status, inflammation

## Abstract

The upper respiratory tract (URT) microbiome can contribute to the acquisition and severity of respiratory viral infections. The described associations between URT microbiota and severe acute respiratory syndrome coronavirus 2 (SARS-CoV-2) infection are limited at microbiota genus level and by the lack of functional interpretation. Our study, therefore, characterized the URT bacterial microbiome at species level and their encoded pathways in patients with COVID-19 and correlated these to clinical outcomes. Whole metagenome sequencing was performed on nasopharyngeal samples from hospitalized patients with critical COVID-19 (*n* = 37) and SARS-CoV-2-negative individuals (*n* = 20). Decreased bacterial diversity, a reduction in commensal bacteria, and high abundance of pathogenic bacteria were observed in patients compared to negative controls. Several bacterial species and metabolic pathways were associated with better respiratory status and lower inflammation. Strong correlations were found between species biomarkers and metabolic pathways associated with better clinical outcome, especially *Moraxella lincolnii* and pathways of vitamin K_2_ biosynthesis. Our study demonstrates correlations between the URT microbiome and COVID-19 patient outcomes; further studies are warranted to validate these findings and to explore the causal roles of the identified microbiome biomarkers in COVID-19 pathogenesis.

## 1. Introduction

The upper respiratory tract (URT) is the primary portal of entry for the severe acute respiratory syndrome coronavirus 2 (SARS-CoV-2) [1], which has caused the coronavirus disease 2019 (COVID-19) pandemic. Infection with SARS-CoV-2 may cause epithelial barrier dysfunction enhancing inflammatory responses and dysbiosis in the respiratory tract, which may worsen the pathogenic processes [2]. It has been evidenced that the URT microbiota may influence the susceptibility and severity of respiratory viral infections [3]. Co-infections of SARS-CoV-2 with other respiratory viruses and bacteria are well described in COVID-19 patients [4,5,6,7,8,9]. Studies have claimed that bacterial co-infections are more frequently encountered in COVID-19 compared to other viral infections [10,11]. However, this has not been confirmed and any increase in bacterial co-infections may rather be due to, e.g., the length of hospital stay and ventilation time. For other viral infections, the presence of certain bacterial organisms can enhance viral acquisition and replication, thereby worsening the respiratory disease [12]. It is therefore of high interest to investigate the URT microbiome and its potential contribution to COVID-19 outcome.

Previous studies have reported relationships between URT microbiota and SARS-CoV-2 infection as well as severity of COVID-19 [13,14,15,16,17,18]. However, most such studies lacked functional interpretation, and taxonomic resolution was limited at genus level using 16S rRNA gene sequencing. The role of the URT microbiome in COVID-19 outcome remains, thus, to be elucidated. Herein, we performed a shotgun whole metagenome sequencing on nasopharyngeal (NP) samples collected from 37 SARS-CoV-2-infected patients and 20 SARS-CoV-2-negative individuals.

## 2. Materials and Methods

### 2.1. Study Design, Collection of Samples and Clinical Data

Nasopharyngeal (NP) specimens were obtained from 37 patients with COVID-19 hospitalized between 27 March 2020 and 18 May 2020 at Karolinska University Hospital, Stockholm, Sweden. All patients had pulmonary infiltrates on chest radiography and eventually received invasive mechanical ventilation at the intensive care unit, thereby being defined as critically ill patients. The SARS-CoV-2 infection was detected using a two-target real-time reverse-transcriptase polymerase chain reaction (RT-PCR) targeting the E gene and RdRp or ORF1 or N2 (*in-house*: RdRp; Cobas: ORF1; GeneXpert: N2). The SARS-CoV-2 load was semiquantified using cycle threshold (Ct) values. NP samples from 20 adults who tested negative for SARS-CoV-2 by RT-PCR were included as negative controls.

Demographic, epidemiological, clinical, and laboratory data of the COVID-19 patients were extracted from the electronic medical records. Clinical and laboratory variables, commonly used as predictors of COVID-19 outcome, were collected within 24 h after the sampling except in three patients. These variables were respiratory rate, oxygen saturation (SpO2), need for supplemental oxygen, PaO2/FiO2 ratio, respiratory Sequential Organ Failure Assessment (SOFA) score, and levels of inflammatory markers including C-reactive protein (CRP), lymphocytes, D-dimer, ferritin, and interleukin-6. Antibiotic use within 3 months prior to sampling was recorded (Table 1 and Supplementary Table S1). The SARS-CoV-2-negative individuals were sampled due to clinical or epidemiological suspicions of SARS-CoV-2 infection.

### 2.2. Nucleic Acid Extraction and Shotgun Metagenome Sequencing

The genomic DNA (gDNA) of the NP samples were extracted by the standardized International Human Microbiota Standards (IHMS) Protocol Q (http://www.microbiome-standards.org, accessed on 26 January 2021) [19] with some modifications. Sequencing libraries were prepared with the Nextera DNA Flex kit (Illumina, CA, USA) following the manufacturer’s instructions. Libraries were normalized with Qubit assay, and then sequenced on NovaSeq6000 (NovaSeq Control Software 1.7.0/RTA v3.4.4) with a 151nt (Read1)-10nt(Index1)-10nt(Index2)-151nt(Read2) setup using ‘NovaSeqXp’ workflow in ‘S4′ mode flowcell. The Bcl to FastQ conversion was performed using bcl2fastq_v2.20.0.422 from the CASAVA software suite. The quality scale used is Sanger/phred33/Illumina 1.8+.

### 2.3. Metagenomics Analysis

The raw sequencing data were pre-processed using our *in-house* bioinformatics pipeline as described previously [20]. Briefly, the adapter and low-quality reads (a quality score of less than Q30) were removed using Trim galore (v0.6.4) (https://www.bioinformatics.babraham.ac.uk/projects/trim_galore/, accessed on 30 March 2021). After the quality trimming, Bowtie2 (v2.3.5.1) [21] was used in combination with SAMtools (v1.19) [22] and BEDtools (v2.29.2) [23] to identify and remove human DNA sequences. The non-human reads were then used for downstream analysis. The bacterial taxonomic assignment and abundance estimation was conducted with MetaPhlAn 3.0 [24] using default parameters. Functional profiling was performed using the HMP Unified Metabolic Analysis Network 3 (HUMAnN 3.0), which quantifies gene families and microbial pathways in microbial community from metagenomic sequencing data [25].

### 2.4. Statistical and Correlation Network Analysis

Alpha diversity of bacterial communities was assessed with microbial richness (number of detected taxa), Shannon and Simpson diversity indices using R function estimate_richness. Differences in alpha diversity between groups were assessed by testing the significance of these indexes using Wilcoxon rank sum test. Beta diversity was measured by Bray–Curtis and weighted UniFrac distances using R package Phyloseq (v1.30.0) [26]. Samples were clustered according to bacterial composition using non-metric multidimensional scaling (NMDS) approach with Bray–Curtis distance in Phyloseq (v1.30.0). Permutational multivariate analysis of variance (PERMANOVA) was performed to test the differences in bacterial composition between groups using vegan package (Adonis function) [27] using a Bray–Curtis dissimilarity method. Given the small sample size, different methods were used to determine and verify specific differences in bacterial taxa and metabolic pathways between groups. In addition to Wilcoxon rank sum test, LEfSe algorithm [28] was used to identify specific bacterial taxa and metabolic pathways as taxonomic and functional biomarkers. Kruskal–Wallis test was used to process the dataset with LEfSe alpha values set at 0.05. The threshold used to consider a discriminative feature for the logarithmic linear discriminant analysis (LDA) score was set at >2.

Correlation analyses were performed using the Spearman’s rank correlation coefficient rho (library ‘‘psych’’, function ‘‘corr.test’’). Correlation network of bacterial species, pathways, and clinical parameters was generated based on the Spearman’s correlation coefficient. The input variables were species biomarkers, metabolic pathways, and clinical markers reflecting COVID-19 outcome. The integration network was constructed using R package bnlearn [29], and only edges of correlation significance test were plotted. Visualization of the network was performed using Cytoscape (v3.6.1) [30]. Benjamini–Hochberg correction was used to adjust *p*-values in the case of multiple testing. Due to small sample size and exploratory purpose of this study, factors with adjusted *p*-value below 0.1 were considered statistically significant; whenever no significant association was identified after correction, results for unadjusted analysis were given, where raw *p*-value below 0.05 was considered significant.

### 2.5. Genome Reconstruction and Functional Annotation of Moraxella Lincolnii

To understand the genetic characteristics and functional potential of species biomarker (i.e., *Moraxella lincolnii*) that was found to be associated with clinical outcome, we performed genome reconstruction through metagenome assembly and functional prediction. Briefly, pre-processed reads were mapped to the reference genome of *Moraxella lincolnii* strain CCUG 9405 (GCA_002014765.1) using Bowtie2 (v2.3.5.1). The mapped reads belonging to *Moraxella lincolnii* were extracted using SAMtools (v1.19), then assembled using MEGAHIT v1.1.3 [31] and kmer lengths starting from 21 to 141. For further confirmation, the assemblies were mapped to the reference *Moraxella lincolnii* genome using BLASTn. The draft genome of *Moraxella lincolnii* was further annotated using RAST (Rapid Annotation using Subsystem Technology) Server (https://rast.nmpdr.org/rast.cgi, accessed on 13 July 2021), which provides high-quality gene calling and functional annotation including a mapping of genes to subsystems and metabolic reconstruction [32].

## 3. Results

### 3.1. Patient Data

NP samples from 37 critically ill COVID-19 patients were collected at a median (range) of 7 (2–35) days after symptom onset. The median (range) age of the patients was 61 years (31–75). The majority (*n* = 30/37, 81.1%) were males. The median (range) body mass index (BMI) was 29.96 (19.27–55). Out of the 37 patients, 25 (67.6%) had other comorbidities including hypertension, diabetes, chronic lung disease, ischemic heart disease, heart failure, systemic inflammatory disease, transplanted, dementia, neurologic disease, and malignancy. Symptoms at admission to hospital included fever, cough, shortness of breath, chest pain, gastrointestinal problems, and loss of taste or smell. Six patients received antibiotics prior to sample collection. Twenty-nine patients were discharged, and eight patients were deceased.

The median (range) Ct value of E gene and SARS-CoV-2 specific RdRp/ORF1/N2 gene was 24.8 (13.8–39.2) and 23.9 (13.8–37.8), respectively. The median Ct value of RdRp/ORF1/N2 gene was used to define higher (Ct value ≤ 23.9, *n* = 17) and lower viral load (Ct value > 23.9, *n* = 20). Patients were further divided into different groups based on clinical variables reflecting COVID-19 outcome, i.e., SpO2 (91–100%, *n* = 6; 81–90%, *n* = 12; ≤80%, *n* = 12; supplemental oxygen support, *n* = 7), PaO2/FiO2 ratio (≥300 mm Hg, *n* = 6; 200–299 mm Hg, *n* = 17; <200 mm Hg, *n* = 14), respiratory SOFA score (0–1, *n* = 6; 2, *n* = 17; 3–4, *n* = 14). The characteristics of COVID-19 patients including levels of inflammatory markers CRP, lymphocytes, D-dimer, ferritin, and IL-6 of COVID-19 patients are shown in Table 1 and Supplementary Table S1.

### 3.2. Composition and Alteration of URT Microbiota Taxa in COVID-19 Patients

**Figure 1:**
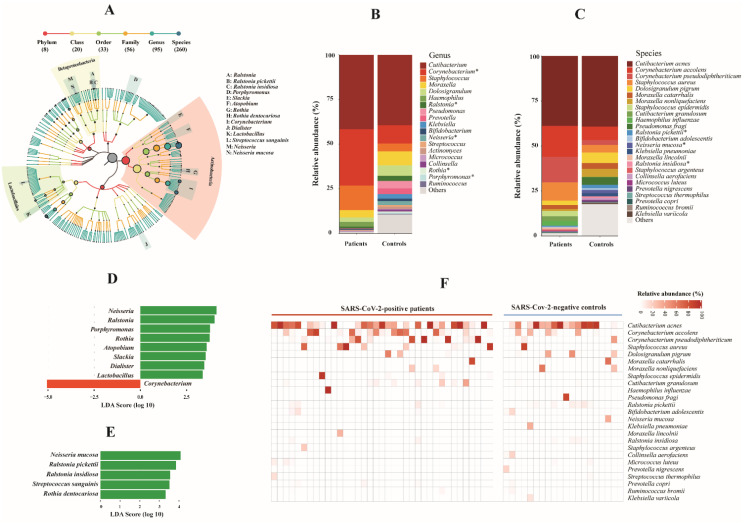
Bacterial microbiota composition in COVID-19 patients and SARS-CoV-2-negative controls. (**A**) Taxonomic tree of identified bacterial taxa. Each dot represents a taxonomic entity. The root of the tree denotes the domain bacteria. From the inner to outer circles, the taxonomic levels range from phylum to species. Different colors of dots indicate different taxonomic levels according to the color key shown. Numbers in parentheses indicate the total number of unique taxa at each taxonomic level. Significantly differentially abundant genera and species between COVID-19 patients and negative controls are labelled with A-N as indicated, more details are shown in Figure 1D,E. The size of each node represents their relative abundance. (**B**,**C**) Bar plots of main bacterial taxa at genus and species levels (average relative abundance > 0.2%) between patients and controls. * Statistically significant difference (Benjamini–Hochberg adjusted *p* < 0.06). (**D**,**E**) Taxonomic biomarkers at genus (**D**) and species level (**E**) identified by linear discriminative analysis (LDA) effect size (LEfSe) analysis between patients (in red) and controls (in green). LDA scores (log 10) for the enriched taxa in controls are represented on the positive scale, while LDA-negative scores indicate enriched taxa in patients. The LEfSe alpha value was set at 0.05, and the threshold used to consider a discriminative feature for the LDA score was set at >2. (**F**) Heat map of abundant bacterial species (average abundance > 0.2%) among individuals between patients and controls. The relative abundance of bacterial species is represented by color gradient as indicated. The species were ordered by decreasing relative abundance.

In total, 260 bacterial species belonging to 95 genera, 59 families, and 33 orders were identified (Figure 1A and Supplementary Table S2). The most abundant bacterial genera were *Cutibacterium*, *Corynebacterium*, and *Staphylococcus*, among which *Corynebacterium* was significantly enriched in COVID-19 patients compared to controls (*p* = 0.0478, Wilcoxon rank sum test). Among other genera with relative abundance above 0.2%, four were significantly decreased in COVID-19 patients (*p* < 0.028, Wilcoxon rank sum test) (Figure 1B). The most abundant bacterial species were *Cutibacterium acnes*, *Corynebacterium accolens*, *Corynebacterium pseudodiphtheriticum*, and *Staphylococcus aureus*. Among 25 species with relative abundance of >0.2%, three were significantly decreased in patients (*p* < 0.018, Wilcoxon rank sum test) (Figure 1C). Using LEfSe, eight out of 95 genera, *Ralstonia*, *Lactobacillus*, *Atopobium*, *Dialister*, *Porphyromonas*, *Slackia*, *Neisseria*, and *Rothia* were significantly decreased in COVID-19 patients, and *Corynebacterium* was significantly enriched in patients (LDA score = 5.052, adjusted *p* = 0.059) (Figure 1D), which was consistent with the Wilcoxon rank sum test. At species level, five species were significantly decreased in COVID-19 patients (LDA score > 3.3, adjusted *p* < 0.056) (Figure 1E). Notably, we observed a high abundance of respiratory bacteria that commonly cause pneumonia in critically ill COVID-19 patients, e.g., *Staphylococcus aureus*, *Haemophilus influenzae*, and *Moraxella catarrhalis* (Figure 1F).

### 3.3. Distinct URT Microbiota Diversity in COVID-19 Patients

**Figure 2:**
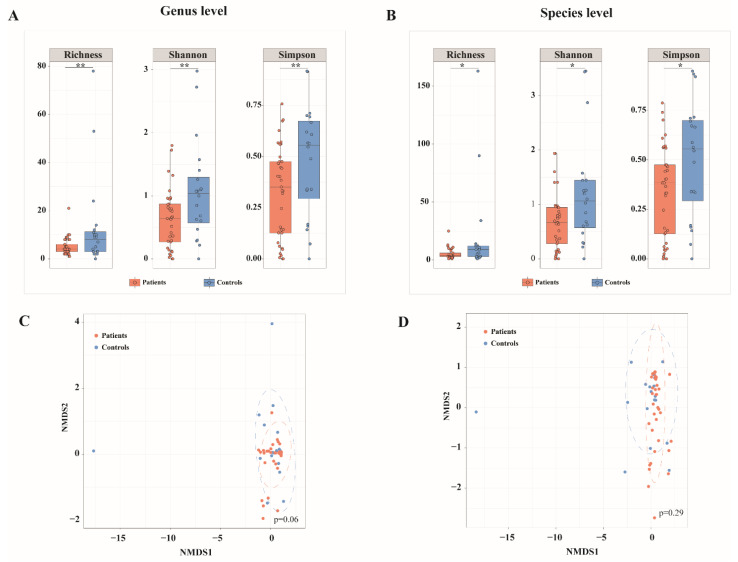
Differences in bacterial microbiota diversity between COVID-19 patients and SARS-CoV-2-negative controls. (**A**,**B**) Alpha diversity in COVID-19 patients and SARS-CoV-2-negative controls at bacterial genus (**A**) and species (**B**) level assessed by microbial richness, Shannon and Simpson diversity indices. (**C**,**D**) Non-metric multidimensional scaling (NMDS) based on Bray–Curtis distance of bacterial composition at genus (**C**) and species (**D**) level between patients and controls. ** Benjamini–Hochberg adjusted *p* < 0.05, * Benjamini–Hochberg adjusted *p* < 0.1.

A significant reduction in alpha diversity of bacterial microbiota at genus level was found in NP samples from COVID-19 patients compared to those from SARS-CoV-2 negative controls, as measured by the microbial richness (adjusted *p* = 0.045, Wilcoxon rank sum test), Shannon and Simpson diversity indices (adjusted *p* = 0.034, Wilcoxon rank sum test) (Figure 2A). Similarly, a decrease in microbial richness, Shannon and Simpson indices of alpha diversity at species level was observed in COVID-19 patients (adjusted *p* < 0.1, Wilcoxon rank sum test) (Figure 2B). No significant difference in beta diversity of bacterial microbiota was found between samples from patients and controls at genus or species level, as assessed with Bray–Curtis and weighted UniFrac dissimilarities (data not shown). NMDS based on Bray–Curtis distance showed no significant separation between patients and controls at genus or species levels (*p* > 0.05, PERMANOVA); however, controls were more diversely distributed than COVID-19 patients (Figure 2C,D).

To assess the potential effect of antibiotics on differences in the bacterial microbiota between groups, we excluded the six COVID-19 patients who had received antibiotics within three months prior to sampling. Similarly, we observed that the alpha diversity of bacterial microbiota at species level in COVID-19 patients was marginally significantly decreased compared to controls, as assessed with Shannon and Simpson diversity indices (*p* = 0.056 and 0.054, respectively, Wilcoxon rank sum test) (Supplementary Figure S1A), while no difference in beta diversity was found between patients and controls (Supplementary Figure S1B,C). The differentially abundant bacterial species between 31 patients who had not been given antibiotics and controls were consistent with those observed between all patients and controls with two exceptions (Supplementary Figure S1D). As information about the use of antibiotics was unavailable in SARS-CoV-2-negative individuals, we did not adjust this factor in subsequent comparative analyses performed between patients and controls.

Given that the bacterial microbiota diversity was decreased in COVID-19 patients, we were interested to determine if samples with higher viral load (Ct value ≤ 23.9, *n* = 17) showed decreased microbiota diversity compared to those with lower viral load (Ct value > 23.9, *n* = 20), but no significant difference was observed (data not shown). No significant correlation was observed between the abundance of bacterial species and Ct values of the SARS-CoV-2 specific gene.

### 3.4. Bacterial Microbiota Associated with Clinical Outcome in COVID-19 Patients

**Figure 3:**
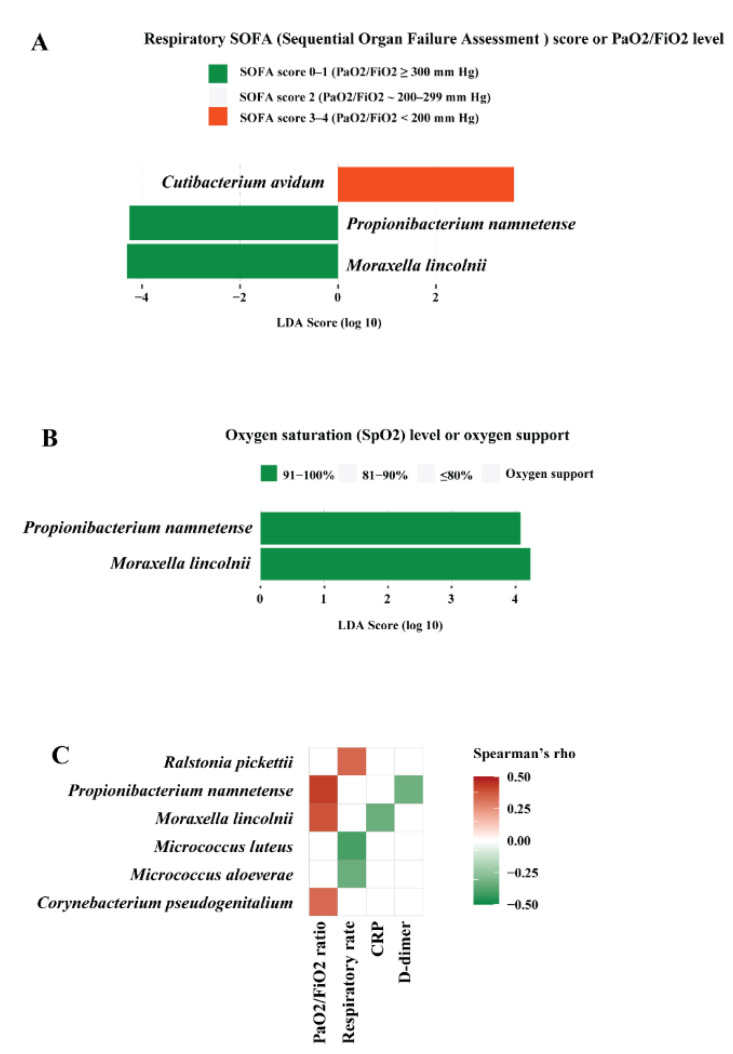
Bacterial microbiota in correlation to clinical outcomes in COVID-19 patients. (**A**) Bacterial species biomarkers associated with respiratory SOFA (Sequential Organ Failure Assessment) score or PaO2/FiO2 level. (**B**) Bacterial species biomarkers associated with oxygen saturation (SpO2) level or oxygen support. The biomarkers were identified by linear discriminative analysis (LDA) effect size (LEfSe) analysis. LDA scores (log 10) for the enriched species in a given group are represented with colors as shown. The LEfSe alpha value was set at 0.05, the threshold used to consider a discriminative feature for the LDA score was set at >2. (**C**) Correlation between bacterial species and markers of respiratory and inflammatory status. Spearman’s correlation rho values are represented by color gradient as indicated (red is for positive, green is for negative correlation). Only correlations with *p* < 0.05 are shown on the plots.

Patients were categorized into groups based on respiratory and inflammatory status reflecting the severity of COVID-19 outcome, as mentioned above. The association between bacterial microbiota and the clinical parameters was analysed by LefSe and Spearman’s correlation analyses. LEfSe showed that *Moraxella lincolnii* and *Propionibacterium namnetense* were enriched in patients who had the highest PaO2/FiO2 ratio (≥300 mm Hg) or lowest respiratory SOFA score 0–1 (*Moraxella lincolnii*: LDA score = 4.307, adjusted *p* = 0.079; *Propionibacterium namnetense*: LDA score = 4.255, adjusted *p* = 0.0089, respectively) (Figure 3A). *Moraxella lincolnii* and *Propionibacterium namnetense* were also enriched in patients with the highest SpO2 91–100% (LDA score = 4.234, *p* = 0.014; LDA score = 4.077, adjusted *p* = 0.026, respectively) (Figure 3B). Spearman’s correlation corroborated the association between microbiota and clinical markers obtained by LefSe. Several bacterial species correlated to certain clinical markers (Figure 3C), among which, *Moraxella lincolnii* and *Propionibacterium namnetense* correlated positively to PaO2/FiO2 ratio, while inversely to inflammation marker CRP and D-dimer, respectively (*p* < 0.05). These data imply that *Moraxella lincolnii* and *Propionibacterium namnetense* may be associated with improved clinical outcome, i.e., better respiratory status and lower inflammation level.

### 3.5. Functional Pathways Associated with Clinical Outcome in COVID-19 Patients

**Figure 4:**
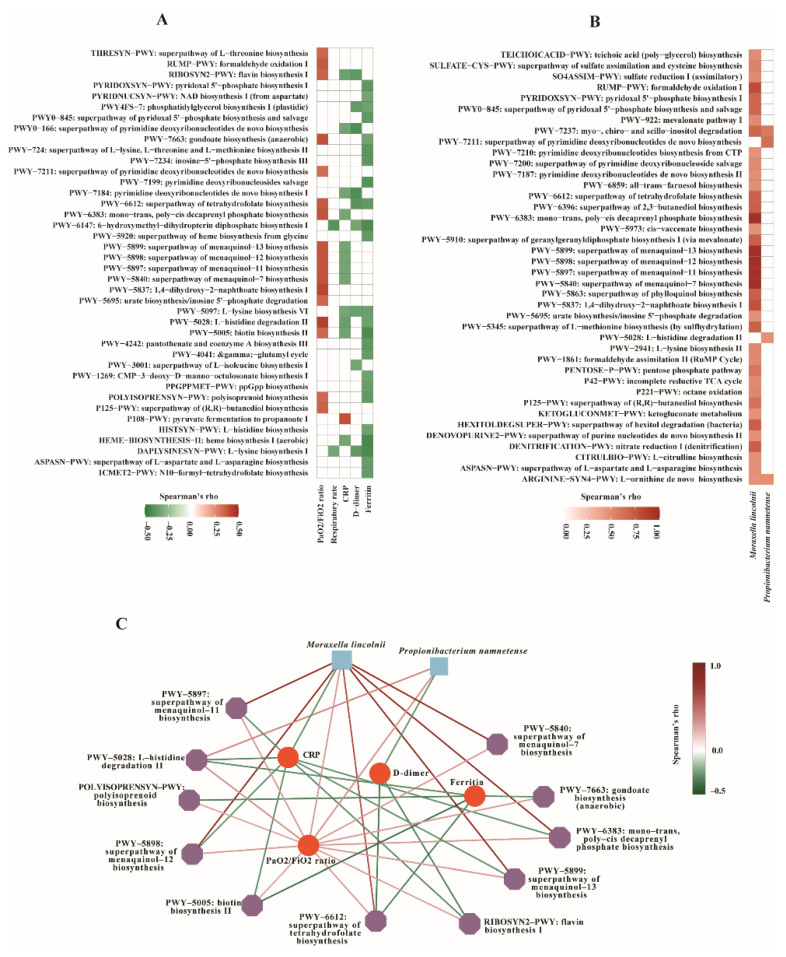
Correlation analysis. Correlations between (**A**) metabolic pathways and clinical markers, (**B**) metabolic pathways and bacterial species biomarkers associated with better clinical outcome. Spearman’s correlation rho values are represented by color gradient as indicated (red is for positive, green is for negative correlation). Only correlations with *p* < 0.05 are displayed. (**C**) Correlation network of bacterial species, metabolic pathways, and clinical markers. The nodes are two bacterial species biomarkers (blue squares), metabolic pathways (purple polygons), and clinical markers (red circles). Two nodes are connected if their Spearman’s correlation is significant (*p* < 0.05). The edge color indicates correlation between subjects (red: positive, green: negative). Spearman’s correlation rho values are represented by color gradient as indicated.

Functional analysis was performed to understand the potential role of the bacterial microbiota in COVID-19 outcome. Intriguingly, several pathways correlated positively to PaO2/FiO2 ratio, while inversely to at least one inflammatory marker, i.e., CRP, D-dimer, or ferritin in COVID-19 patients (*p* < 0.05, Spearman’s correlation) (Figure 4A). These included: (i) superpathways of menaquinol-7, menaquinol-11, menaquinol-12, and menaquinol-13 biosynthesis; (ii) mono-trans, poly-cis decaprenyl phosphate biosynthesis pathway; (iii) superpathway of tetrahydrofolate biosynthesis; (iv) gondoate biosynthesis (anaerobic); (v) flavin biosynthesis I; (vi) biotin biosynthesis II; (vii) L-histidine degradation II; (viii) polyisoprenoid biosynthesis. Interestingly, most of these pathways belong to the same superclass ‘vitamin biosynthesis’; in particular, the four superpathways of menaquinone biosynthesis are known as vitamin K_2_ biosynthesis. These results might indicate a potential role of vitamin K_2_ in better clinical outcome, i.e., better respiratory status and lower inflammation in COVID-19.

Additionally, we observed that the PaO2/FiO2 ratio correlated positively to pathways involved in 1,4-dihydroxy-2-naphthoate biosynthesis I, formaldehyde oxidation I, etc. CRP level correlated inversely to pathways involved in pyrimidine deoxyribonucleotides de novo biosynthesis, heme biosynthesis I (aerobic), etc., while they correlated positively to pyruvate fermentation to propanoate I. The serum ferritin level correlated inversely to pathways involved in heme biosynthesis I (aerobic), 6-hydroxymethyl-dihydropterin diphosphate biosynthesis I, L-lysine biosynthesis I, etc. D-dimer correlated inversely to several functional pathways (*p* < 0.05, Spearman’s correlation) (Figure 4A).

### 3.6. Correlations between Bacterial Microbiota and Metabolic Pathways Contributing to Clinical Outcome

To assess the correlations between bacterial microbiota and metabolic pathways contributing to respiratory and inflammatory status, Spearman’s correlation analysis was performed between bacterial species and functional pathways in COVID-19 patients. Interestingly, we observed very strong correlations between species and pathway biomarkers that were associated with clinical markers. *Moraxella lincolnii* correlated strongly to superpathways of menaquinol-7, menaquinol-11, menaquinol-12, and menaquinol-13 biosynthesis (rho = 1, adjusted *p* < 2.2 × 10^−16^); mono-trans, poly-cis decaprenyl phosphate biosynthesis (rho = 0.998, adjusted *p* = 2.33 × 10^−43^); formaldehyde oxidation I (rho = 0.805, adjusted *p* = 2.50 × 10^−7^); 1,4-dihydroxy-2-naphthoate biosynthesis I (rho = 0.781, adjusted *p* = 1.23 × 10^−6^), etc. (Figure 4B). Most of these pathways, as mentioned above, belong to the same superclass ‘vitamin biosynthesis’. *Propionibacterium namnetense* correlated positively to the superpathway of pyrimidine deoxyribonucleotides de novo biosynthesis (rho = 0.657, adjusted *p* < 0.0001), and L-histidine degradation II (rho = 0.478, adjusted *p* = 0.0425) (Figure 4B).

Correlation network analysis was performed to unravel any interaction among two species biomarkers, metabolic pathways, and clinical markers reflecting COVID-19 outcome. Results demonstrated species-to-pathways, species-to-clinical markers, and pathway-to-clinical markers interconnections, e.g., *Moraxella lincolnii* to superpathways of menaquinol biosynthesis, *Moraxella lincolnii* to CRP and the PaO2/FiO2 ratio, and superpathways of menaquinol biosynthesis to CRP and the PaO2/FiO2 ratio (Figure 4C). These data highlight the associations between microbiota and metabolic pathways contributing to clinical outcome in COVID-19 patients.

### 3.7. Genomic Feature and Functional Potential of Moraxella Lincolnii

Given that *Moraxella lincolnii* was found to be associated with respiratory and inflammatory status, and that it also correlated very strongly to the metabolic pathways associated with better respiratory and inflammatory status, particularly vitamin K_2_ biosynthesis, we were interested to identify genetic evidence that could support this finding. We therefore performed genome reconstruction and functional annotation of *Moraxella lincolnii* from one sample with a high abundance of this species. Genomic characteristics of *M. lincolnii* are summarized in Supplementary Table S3. Notably, a gene that encodes a key enzyme in menaquinone (vitamin K_2_) biosynthesis was identified in the *M. lincolnii* genome, i.e., *ubiE* (bifunctional demethylmenaquinone methyltransferase/2-methoxy-6-polyprenyl-1,4-benzoquinol methylase). Additionally, *M. lincolnii* possess genes involved in several crucial metabolic pathways that are known to act in an integrated manner to maintain the balance and organism homeostasis, including genes involved in lipid metabolism, amino acid metabolism, glycolysis, pentose phosphate pathway, etc. (Supplementary Table S3b and Supplementary Figure S2). These results support our hypothesis that *M*. *lincolnii* contributes to vitamin K_2_ biosynthesis and other metabolic pathways, thereby, possibly being associated with better clinical outcome in COVID-19 patients. Further in vitro studies are in need to validate the effect of *M. lincolnii* in vitamin K_2_ biosynthesis and other biological functions that may play a beneficial role in COVID-19.

## 4. Discussion

In this study, using shotgun whole metagenome sequencing, we characterized the upper respiratory microbiome profile in critically ill COVID-19 patients and correlated the findings to viral load, and respiratory and inflammatory status. No association was found between SARS-CoV-2 loads and bacterial microbiota diversity or differentially abundant bacterial taxa, which is in line with a previous report using metagenome sequencing [14]. The viral load in the URT is highest early in the disease course [33,34], and since our sampling occurred a median of 7 days after symptom onset, the peak of viral replication had most probably passed. Intriguingly, in the COVID-19 patients, several bacterial species were associated with markers of both respiratory and inflammatory status; in particular, *Moraxella lincolnii* and *Propionibacterium namnetense* were correlated to better respiratory status and low inflammation. *M. lincolnii*, a poorly characterized bacterium isolated from the human respiratory tract [35], was found in two of the COVID-19 patients who showed high viral load but with very low levels of inflammatory markers and normal respiratory conditions, implying that *M. lincolnii* might increase the host immunity against SARS-CoV-2. We acknowledged that this hypothesis remains to be confirmed since only two patients harbored *M. lincolnii*. It is noteworthy that a recent study has indeed suggested a protective role of *M. lincolnii* in respiratory health status [36]. Moreover, *M. lincolnii* has recently been shown to exhibit strong inhibitory activity against nasal *S. aureus*, mediated by proteins fitting the profile of antimicrobial peptides (AMPs) [37]. AMPs are known to exhibit antiviral and immunomodulatory properties [38], which possibly could contribute to improved patient outcomes in viral respiratory infections. Research on the recently identified *P. namnetense* [39] is also very rare, our data appeal for further in-depth studies to investigate the potential role of these two species in COVID-19 outcomes.

To date, the most probable hypothesis on how the respiratory microbiome could influence viral respiratory infections relies on the immunological properties of microbes inhabiting the respiratory tract [3]. At functional level, we found that several metabolic pathways correlated to both better respiratory status and to lower inflammation level. Interestingly, most of these pathways are involved in vitamin biosynthesis, particularly four superpathways of menaquinone (vitamin K_2_) biosynthesis. Several other pathways that correlated to better respiratory status or/and lower inflammation level belonged to pathway superclass ‘Cofactor, Carrier, and Vitamin Biosynthesis’, such as 1,4-dihydroxy-2-naphthoate biosynthesis I, which is the naphthalenic intermediate in the biosynthesis of vitamin K_2_ [40]. Our results may, thus, indicate a potential role of vitamin K_2_ in improving clinical outcome in COVID-19. This is supported by the reported correlations between vitamin K deficiency and severe COVID-19 outcome [41,42]. Vitamin K is associated with an impaired production of inflammatory cytokines and plays an important role in immunomodulation [43,44]. In addition to attenuating the excessive production of proinflammatory cytokines [45], vitamin K may protect the integrity of the alveolar-capillary membrane [46], thereby, possibly improving respiratory status in COVID-19 patients as we observed.

Another remarkable finding was the strong correlation between the two species biomarkers (*M. lincolnii*, *P. namnetense*) and metabolic pathways associated with better respiratory status and lower inflammation level. In particular, *M. lincolnii* correlated strongly to pathways involved in vitamin K_2_ biosynthesis. Vitamin K naturally occurs in two biologically active forms, K_1_ and K_2_; of these, vitamin K_2_ is predominantly of bacterial origin [47,48]. In order to confirm this and gain insights into the functions of *M. lincolnii*, we reconstructed the draft genome of *M. lincolnii* from metagenome data and performed functional annotation. Strikingly, we found that *M. lincolnii* carried the gene encoding ‘bifunctional demethylmenaquinone methyltransferase/2-methoxy-6-polyprenyl-1,4-benzoquinol methylase’, an enzyme catalyzing the last step in menaquinone (vitamin K_2_) biosynthesis. *M. lincolnii* possess additional genes involved in several metabolic pathways that have been suggested to be associated with COVID-19, e.g., lipid and amino acid metabolism, heme biosynthesis, glycolysis, pentose phosphate pathway, etc. [49,50]. Our data indicate a great need for in vitro research to validate the effects of *M. lincolnii* in vitamin K_2_ biosynthesis and other metabolic processes that may play beneficial roles in COVID-19 outcome.

Our study demonstrated a reduced bacterial microbiota diversity in the critical COVID-19 patients compared to the SARS-CoV-2-negative individuals. Some respiratory pathogens, particularly pneumonia-causing bacteria, e.g., *Staphylococcus aureus* and *Haemophilus influenzae,* were abundant in the critically ill COVID-19 patients, highlighting the possibility that co-infections with such pathogens may contribute to severe clinical outcome. In contrast, a significant reduction in respiratory commensals, e.g., *Neisseria mucosa*, *Ralstonia pickettii,* was found in the COVID-19 patients. Such reductions in healthy commensals might contribute to the susceptibility to and severity of SARS-CoV-2 infection, as suggested in other viral infections [3], although the causal relationship between URT microbiome alteration and SARS-CoV-2 infection warrants further investigation. It should be noted that most of our critically ill COVID-19 patients had well-known risk factors for disease severity, such as age, gender, and comorbidities [51,52], which may be confounders contributing to URT microbiome changes in the patients. Moreover, although the controls were sampled due to suspicions of SARS-CoV-2 infection, we could not rule out potential confounders that may explain the differences in the URT microbiome observed.

This study has limitations. The major flaws were the small sample size and the lack of detailed information of SARS-CoV-2-negative controls; thus, the microbiome changes in COVID-19 patients and microbiome biomarkers identified for clinical outcomes remain to be validated with further studies. Second, only critically ill COVID-19 patients were included, and a single NP sample per patient collected at hospital admission was analysed, our findings may not apply to patients with asymptomatic, mild to moderate COVID-19. Third, although whole metagenome sequencing has obvious advantages, particularly its functional profiling capacity, it suffers from host-derived DNA contamination, which may obscure microbial signatures in low-biomass and highly host-contaminated NP samples. 16S rRNA sequencing should be considered in combination with whole metagenome sequencing to obtain a comprehensive landscape of URT microbial communities and functionality. In spite of these limitations, our study reveals important information for the interpretation of the role of the URT microbiome in SARS-CoV-2 infection. It is noteworthy that the correlations observed in this study do not illustrate a direct causal link between the URT microbiome and SARS-CoV-2 infection as described widely in other microbiome studies [53,54,55]; further in-depth studies are warranted to explore the causal roles of the URT microbiome in the COVID-19 pathogenesis.

In conclusion, our study characterized the URT microbiome in correlation to COVID-19 outcomes. Several bacterial species and metabolic pathways were associated with respiratory and inflammation status in COVID-19 patients. Strong associations were found between two species biomarkers and several pathways that were associated with better clinical outcome; in particular, *Moraxella lincolnii* and pathways involved in vitamin K_2_ biosynthesis. To our knowledge, this is the first study to depict the URT microbiome associated with respiratory status in critical COVID-19 patients. In addition, our study demonstrates a distinct URT microbiome profile in patients with critical COVID-19 compared to non-COVID-19 individuals. These findings aggregately render evidence of the URT microbiome as a possible contributor to COVID-19 outcome. Future in-depth studies are warranted with a larger sample size, serial samples from each patient, samples from other geographic areas, combination of different sequencing techniques, and in vitro assays to elucidate the causal roles of URT microbiome changes in SARS-CoV-2 infection, disease progression, and patient outcomes. This could possibly aid the identification of microbial targets for potential interventions and treatments of COVID-19.

## Figures and Tables

**Table 1:** Characteristics of 37 COVID-19 patients ^#^.

Sample ID	Viral Load ^a^	Antibiotics Use ^b^	Days From Onset ^c^	CRP ^d^ (mg/L)	Ferritin (μg/L)	D-Dimer (mg/L)	Respiratory Rate (No./min)	SpO2 or Oxygen Support	PaO2/FiO2 (mm Hg)	Respiratory SOFA Score
NP-C1	High	No	7	119	1882	6.5	18	91–100%	≥300	0–1
NP-C2	Low	No	2	252	1219	0.58	36	81–90%	200–299	2
NP-C3	Low	Yes	7	177	829	1.1	NA	Oxygen support	<200	3
NP-C4	Low	No	12	208	NA	3.2	24	81–90%	200–299	2
NP-C5	Low	No	8	89	4010	2.8	40	≤80%	200–299	2
NP-C6	Low	No	7	202	2050	2	32	81–90%	200–299	2
NP-C7	High	No	4	41	624	0.67	18	81–90%	200–299	2
NP-C8	High	No	5	158	66	3.6	28	91–100%	≥300	0–1
NP-C9	High	No	14	144	1308	1.06	25	81–90%	200–299	2
NP-C10	High	No	9	195	927	0.7	23	81–90%	200–299	2
NP-C11	Low	No	6	227	1163	2.1	26	Oxygen support	<200	3
NP-C12	High	No	23	38	440	0.3	18	91–100%	≥300	0–1
NP-C13	Low	No	2	138	3592	1.97	35	Oxygen support	<200	4
NP-C14	Low	No	3	318	1167	12.1	30	≤80%	<200	3
NP-C15	Low	No	15	212	2985	2	24	≤80%	<200	3
NP-C16	High	Yes	7	46	1822	0.96	23	Oxygen support	200–299	2
NP-C17	High	Yes	5	41	NA	0.64	24	81–90%	200–299	2
NP-C18	Low	No	5	46	1026	0.5	22	91–100%	≥300	0–1
NP-C19	Low	No	7	143	252	1.68	28	81–90%	200–299	2
NP-C20	Low	No	29	98	366	4.1	32	81–90%	200–299	2
NP-C21	Low	No	7	319	1374	0.51	23	81–90%	200–299	2
NP-C22	Low	No	14	222	1550	1.6	22	≤80%	<200	3
NP-C23	High	No	5	358	2843	0.46	35	≤80%	<200	3
NP-C24	High	No	10	58	3621	1.04	23	≤80%	<200	3
NP-C25	Low	No	5	319	959	1.03	40	≤80%	<200	3
NP-C26	Low	No	7	75	1024	0.34	16	81–90%	200–299	2
NP-C27	High	No	35	260	NA	0.8	45	≤80%	200–299	2
NP-C29	Low	No	5	99	1562	0.9	22	≤80%	<200	3
NP-C31	High	No	7	316	1361	1.08	35	≤80%	<200	3
NP-C32	High	No	3	256	1914	1.05	40	Oxygen support	<200	4
NP-C34	High	No	3	54	810	0.46	18	91–100%	≥300	0–1
NP-C35	Low	Yes	6	190	1601	0.77	30	≤80%	200–299	2
NP-C37	High	No	2	41	693	0.69	26	91–100%	≥300	0–1
NP-C38	High	No	3	42	40	0.25	28	≤80%	200–299	2
NP-C39	High	No	3	30	314	4.1	26	81–90%	200–299	2
NP-C40	Low	Yes	19	12	453	2.3	NA	Oxygen support	<200	4
NP-C42	Low	Yes	14	368	1793	5.3	30	Oxygen support	<200	4

^#^ The laboratory and clinical parameters represent status at sampling. Antibiotic use within 3 months prior to sampling was recorded. Other patient metadata are shown in Supplementary Table S1. ^a^ The Ct values of E gene and SARS-CoV-2 specific gene RdRp/ORF1/N2 are shown in Supplementary Table S1. ^b^ Antibiotics received are shown in Supplementary Table S1. ^c^ Days from onset are defined as the number of days from the onset of initial symptom to the time of sample collection. The date of initial symptom and sampling are present in Supplementary Table S1. ^d^ C-reactive protein. NA: unavailable.

## Data Availability

The raw shotgun metagenome sequencing data have been submitted to the NCBI Sequence Read Archive under the accession number PRJNA781460.

## Supplementary Materials

The following supporting information can be downloaded at: https://www.mdpi.com/article/10.3390/biomedicines10050982/s1, Table S1. Clinical and laboratory metadata of COVID-19 patients (.xlsx). Table S2. Bacterial species identified in this study and their relative abundance in each sample (.xlsx). Table S3. Genomic characteristics and functional annotation of Moraxella lincolnii (.xlsx). Figure S1. Differences in bacterial microbiota diversity and differentially abundant species between 31 COVID-19 patients who did not take antibiotics prior to sampling and 20 SARS-CoV-2-negative controls. (A) Alpha diversity between COVID-19 patients and SARS-CoV-2-negative controls at bacterial species level assessed by richness, Shannon and Simpson diversity indices. (B,C) Bray–Curtis dissimilarity and non-metric multidimensional scaling (NMDS) based on Bray–Curtis distance of bacterial species composition between patients and controls. (D) Bacterial species biomarkers identified by linear discriminative analysis (LDA) effect size (LEfSe) analysis between 31 COVID-19 patients (in red) and 20 controls (in green). LDA scores (log 10) for the enriched species in controls are represented on the positive scale, while LDA-negative scores indicate enriched species in patients. The LEfSe alpha values was set at 0.05, the threshold used to consider a discriminative feature for the LDA score was set at >2. Figure S2. Subsystem distribution of *Moraxella lincolnii* identified in this study. The functional annotation and subsystem categorization was conducted using RAST server.
