# Supplementary material for: Characterization of the Upper Respiratory Bacterial Microbiome in Critically Ill COVID-19 Patients
Source: Biomedicines. 2022 Apr 23;10(5):982. doi: 10.3390/biomedicines10050982 (PMC9138573; doi:10.3390/biomedicines10050982)
Supplement: Supplementary file 1 [file biomedicines-10-00982-s001.zip › biomedicines-1672409-supplementary/Supplementary Materials/Supplementary Figure S2.pdf]

Figure S2

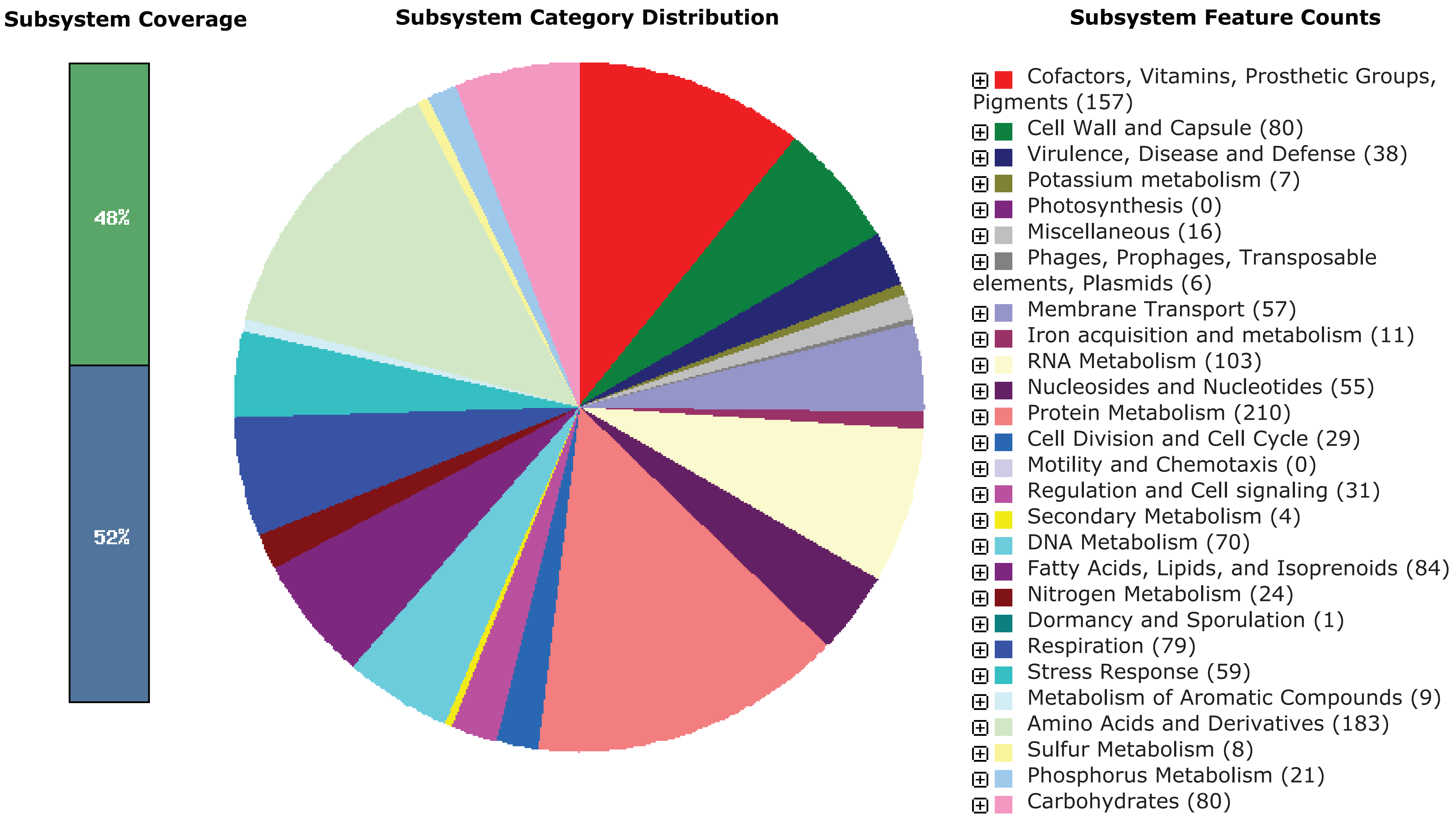

Subsystem Coverage
48%
52%
Subsystem Category Distribution
Subsystem Feature Counts
Cofactors, Vitamins, Prosthetic Groups, Pigments (157)
Cell Wall and Capsule (80)
Virulence, Disease and Defense (38)
Potassium metabolism (7)
Photosynthesis (0)
Miscellaneous (16)
Phages, Prophages, Transposable elements, Plasmids (6)
Membrane Transport (57)
Iron acquisition and metabolism (11)
RNA Metabolism (103)
Nucleosides and Nucleotides (55)
Protein Metabolism (210)
Cell Division and Cell Cycle (29)
Motility and Chemotaxis (0)
Regulation and Cell signaling (31)
Secondary Metabolism (4)
DNA Metabolism (70)
Fatty Acids, Lipids, and Isoprenoids (84)
Nitrogen Metabolism (24)
Dormancy and Sporulation (1)
Respiration (79)
Stress Response (59)
Metabolism of Aromatic Compounds (9)
Amino Acids and Derivatives (183)
Sulfur Metabolism (8)
Phosphorus Metabolism (21)
Carbohydrates (80)
