# Supplementary figures and images for: Characterization of the Upper Respiratory Bacterial Microbiome in Critically Ill COVID-19 Patients
Source: Biomedicines. 2022 Apr 23;10(5):982. doi: 10.3390/biomedicines10050982 (PMC9138573; doi:10.3390/biomedicines10050982)

Figure S1

A

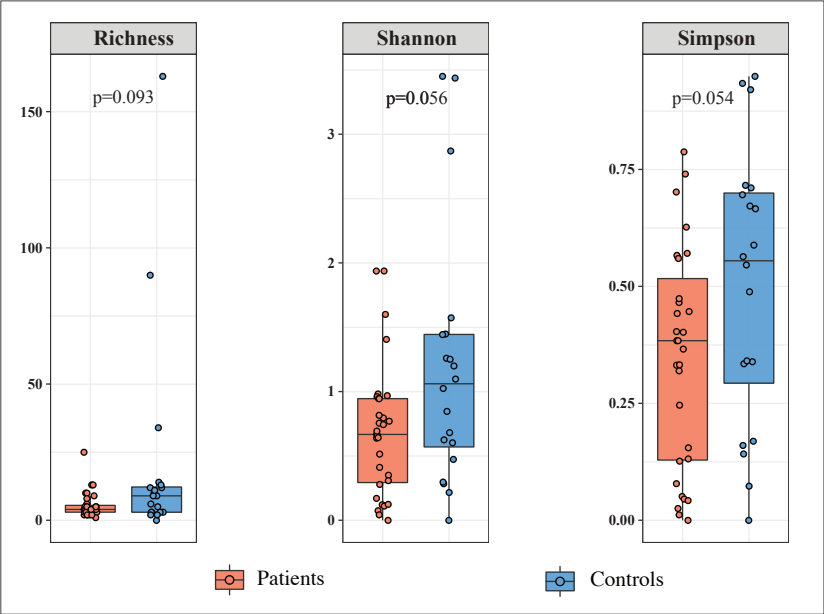

B

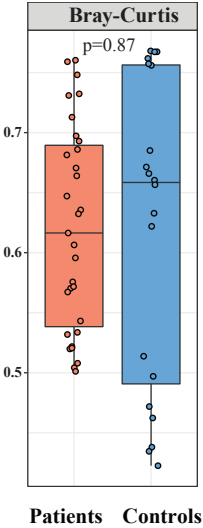

C

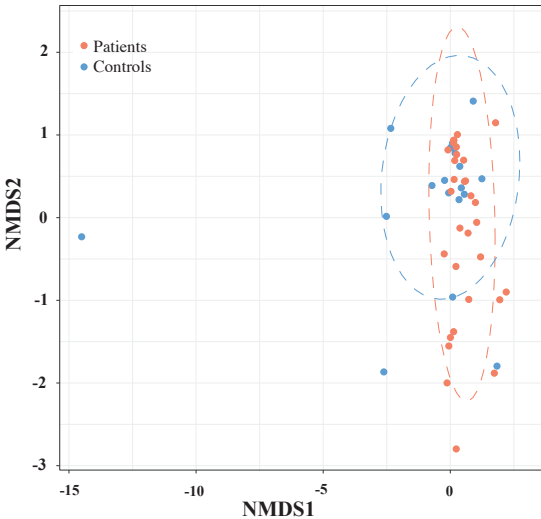

D

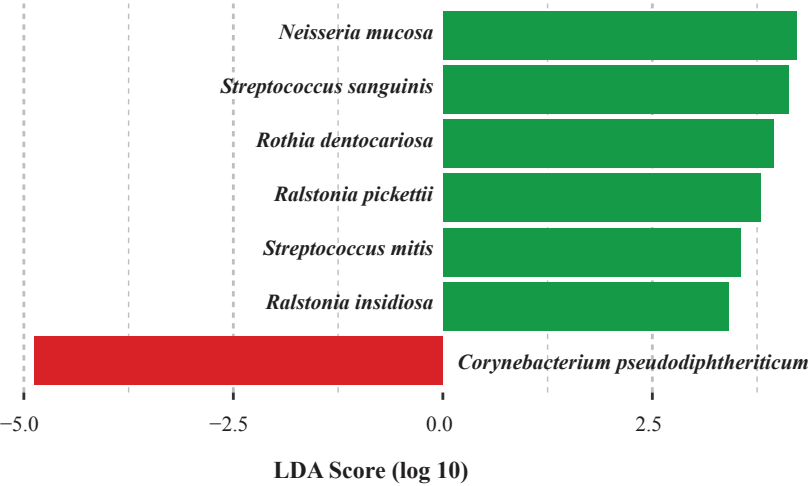

Supplement: Supplementary file 1 [file biomedicines-10-00982-s001.zip › biomedicines-1672409-supplementary/Supplementary Materials/Supplementary Figure S1.pdf]
